# Challenging Surgical Treatment of Displaced Articular Tibial Plateau Fractures: Do Early Knee Radiographic Features Have a Predictive Value of the Mid‐Term Clinical Functional Outcomes?

**DOI:** 10.1111/os.12577

**Published:** 2019-11-22

**Authors:** Carlo Biz, Giacomo Maso, Marisa Gambato, Elisa Belluzzi, Assunta Pozzuoli, Marta Favero, Marco Vigo, Pietro Ruggieri

**Affiliations:** ^1^ Orthopaedic Clinic, Department of Surgery, Oncology and Gastroenterology University of Padova, Padova Italy; ^2^ Musculoskeletal Pathology and Oncology Laboratory, Department of Surgery, Oncology and Gastroenterology University of Padova Padova Italy; ^3^ Rheumatology Unit, Department of Medicine University of Padova Padova Italy

**Keywords:** Articular fractures, Open reduction and internal fixation, Post‐traumatic osteoarthritis, Tibial plateau fractures, Tibial proximal fractures

## Abstract

**Objective:**

To evaluate mid‐term radiographic and functional outcomes of tibial plateau fracture (TPF) patients treated with the open reduction internal fixation (ORIF) technique and to find predictive factors of clinical outcomes.

**Methods:**

A retrospective, single‐center study was performed enrolling a consecutive series of patients with diagnosis of TPF. All subjects were treated by ORIF between January 2010 and December 2015 at our level‐1 healthcare trauma center. The inclusion criteria were: age between 18 and 75 years; ORIF technique used for articular TPF, type 41‐B and 41‐C, isolated or with associated injuries. The patients were divided in two groups, according to fracture patterns and compared. Their characteristics, radiographic and clinical outcomes were recorded. Radiographs 12 months after surgery were evaluated for reduction and alignment, and radiographs at 24 months to describe post‐traumatic osteoarthritis (PTOA). Functional outcomes were assessed using the visual analog scale (VAS), the Short Form 36 (SF‐36), the knee injury and osteoarthritis outcome score (KOOS), and the American Knee Society score (AKSS) questionnaires. Return to work and sport activities were also investigated. Univariate and multivariate analysis were performed, and the statistical significance was defined as two‐tailed *P* < 0.05.

**Results:**

Forty‐five patients were included, 29 males and 16 females; the mean age was 54.5 years. The mean follow‐up was 57.18 months (range, 26–94). There were AO 41‐B fractures (partial articular fractures) in more than half of the patients (66.67%), while the remaining 15 had AO 41‐C fractures (complete articular fractures). The sub‐type AO 41‐B3 was the most common, reported in 62.22% of patients. The mean KOOS score was 69.0. Mean AKSS and SF‐36 PCS scores were 79.0 and 41.4, respectively. There were significant relationships between age and functional results (KOOS ADL, Sport, QoL, and SF‐36 PCS) and between BMI and KOOS Pain, ADL, Sport, and QoL. No differences were found between the two types of fractures regarding quality of reduction and alignment. AO 41‐C TPF tend to develop PTOA more frequently with respect to 41‐B fractures, while type AO 41‐C TPF had the worst clinical outcomes. We found that the presence of an articular step‐off and the malalignment of the tibial axis after surgery were predictive of daily pain felt by patients. PTOA was predictive of a worse AKSS. The overall complication rate was 13.33%: 1 superficial wound infection, 1 deep vein thrombosis, and 4 cases of transitory deficit of the common peroneal nerve.

**Conclusion:**

The present study demonstrates that early radiographic features may be predictive for pain perceived by patients at mid‐term follow‐up.

## Introduction

Tibial plateau fractures (TPF) account for 1% of all bone fractures^1^ and 9.2% of tibial fractures^2^. Elsoe *et al*. showed that TPF have an incidence of 10.3 per 100 000 people annually, with AO type 41‐B3 being the most common type (35% of all TPF), followed by AO type 41‐C3 representing 17% of all TPF^3^. These fractures occur more frequently in males than in females, and the highest frequency is between the ages of 40 and 60 years in both men and women^3^. Generally, younger and middle‐aged men (64%) tend to have fractures as a result of high‐energy trauma, such as high‐speed motor vehicle accidents or falls from a considerable height, while older women have low‐energy fractures (35%)^3^. In elderly patients, TPF may also occur due to a simple fall and low‐energy trauma^4^.

As for pilon fractures^5^, TPF treatment is challenging because of the difficulty of reducing and retaining multiple articular fragments, as well as restoring axial alignment and anatomically reconstructing the joint surface combined with knee stability, without compromising soft tissue^6^. Moreover, the involvement of cartilage and subchondral bone lesions, as well as metaphyseal compression of trabecular bone structures, contributes to an uncertain outcome of TPF^6^. According to the Arbeitsgemeinschaft fur Osteosynthesefragen/Orthopedic Trauma Association (AO/OTA) classification system, nonoperative treatment is no longer used because of poor results, except for simple undisplaced and extra‐articular fractures^7^, ^8^.

The goal of treating TPF is to obtain a stable joint, enabling early mobilization of the knee^9^. A preoperative CT scan for fracture analysis is mandatory, and optimal articular reduction and axial alignment restoration of the knee are necessary to achieve this objective. The functional outcome is shown mainly by the range of motion (ROM), joint stability, and pain^10^. In the current literature, there are few data and there is limited consensus about the correlation of congruent articular reduction, tibial plateau alignment, and functional outcome after surgical treatment and the development of secondary osteoarthritis (OA) in patients with TPF^11^.

Today, TPF treatment procedures can include open reduction and internal fixation (ORIF), closed reduction and percutaneous fixation, arthroscopic‐assisted reduction and internal fixation (AARIF), or external fixation techniques. Treatment strategies for complex TPF may include the combination of external and internal fixation. For example, the use of a wire external fixator allows fracture stabilization with minimal direct approach and a small surgical incision^10^, ^12^, ^13^, ^14^. Regarding less severe fractures, closed reduction and percutaneous fixation by cannulated screws is characterized by minimal invasiveness and low surgical risk^15^, ^16^. For simple split fracture patterns, indirect reduction and percutaneous screw fixation is recommended^17^. Currently, the open anatomic reduction and internal fixation technique is the most common surgical technique for displaced TPF 18, 19, 20, 21. This technique involves the use of plates, providing increased angular stability, and can be associated with different bone grafting methods. The treatment strategy is dependent on the condition of the soft tissue envelope; a temporary stabilization with external fixation is often required to allow soft tissue restoration, postponing definitive treatment to the optimal time^22^.

Despite anatomical joint reconstruction, TPF are considered a major risk factor for post‐traumatic osteoarthritis (PTOA)^23^, which develops in 9%–44% of injured patients^23^. An initial trauma of the tibial plateau cartilage may be the first cause of the development of osteoarthritic problems.

Interestingly, Wasserstein *et al*. reported that 10 years after TPF surgery, 7.3% of the patients had a total knee arthroplasty. This corresponds to a 5.3 time increase in likelihood compared to a matched group from the general population^24^. The incidence of PTOA following TPF varies in the current literature^23^, ^25^, and there is still limited data at medium‐term following operative treatments of TPF.

As most of the patients with TPF are active and highly demanding, an important issue is the incidence of OA, which can markedly impact the quality of life and affect the return both to sports and work activities. For these reasons, patients should be informed of the risk of developing PTOA, which could require future surgical intervention.

Hence, the primary aim of this retrospective study was to evaluate the mid‐term radiographic and functional outcomes of patients surgically treated by ORIF using locking plates for partial articular (AO 41 type B) and complete articular (AO 41 type C) TPF. The second aim was to investigate whether radiographic features 12 months after surgery and the development of PTOA at 24 months were predictive of clinical‐functional outcome at last follow‐up.

## Materials and Methods

### *Patient Selection*

Between January 2010 and December 2015, 63 consecutive patients with the same number of TPF underwent ORIF surgical procedures at our level‐1 healthcare trauma center, a 1572‐bed multi‐disciplinary and multi‐specialty regional university teaching hospital. All subjects participating in this retrospective and experimental study received a thorough explanation of the risks and benefits of inclusion and gave their oral and written informed consent to publish the data. The study was performed in accordance with the ethical standards of the 1964 Declaration of Helsinki as revised in 2000. The Local Ethical Committee approved the study (Prot. num 32110, Tit.II el. fasc‐141). The patients received a thorough explanation of this study and gave their oral and written informed consent to be included in this analysis.

The inclusion criteria were as follows: (i) patients' aged between 18 and 75 years old at the time of the trauma, admitted to our trauma unit in the selected period; (ii) ORIF technique used for articular TPF, isolated or with associated fractures, type 41‐B and 41‐C, according to Muller‐AO classification^8^; (iii) patients with type 41‐B and 41‐C, divided in two groups and compared; and (iv) patient characteristics, radiographic data, and clinical outcomes (orthopedic visit, visual analog scale [VAS], the short form 36 [SF‐36], knee injury and osteoarthritis outcome score [KOOS]) were recorded. The design of the study was retrospective and single center.

For this study, specific patient exclusion criteria were as follows: (i) pediatric patients; (ii) history of previous foot surgery or trauma; (iii) diagnosis of diabetes mellitus; (iv) cancer patients receiving chemotherapy and radiotherapy or with bone metastases; (v) patients with severe orthopaedic comorbidities (coxarthrosis, rheumatological diseases or psoriatic arthritis, diabetic foot neuropathy, or vascular insufficiency); and (vi) patients with untraceable postoperative radiographs.

### *Surgical Technique*

All patients were operated on by an orthopaedic surgeon of our trauma team (the senior authors) with the help of two residents. A tourniquet was not routinely used during synthesis procedures.

#### *Step 1*

Anesthesia and position: all anesthesiological procedures were performed with an ultrasound‐guided peripheral nerve block with atropine injections in combination with analgosedation. For isolated lateral TPF, a peripheral nerve bi‐block (femoral and sciatic) was used, while in medial or bi‐condylar TPF, an ultrasound‐guided adductor canal block or laryngeal mask airway with deep sedation were also performed. Patients were in supine position on the operating table with the injured knee flexed approximately 60°. Prophylactic cefazolin (2 g) was administered and continued 24 h after surgery, while postoperative antithrombotic therapy (natriumenoxaparin) was started the same evening after the operation until weight‐bearing. During surgery, an image intensifier was used to enable optimal anatomical reduction and alignment. Temporary external fixation was used only when soft tissue conditions precluded definitive internal fixation.

#### *Step 2*

Approach and exposure: for unicondylar lateral TPF, a straight or hockey stick anterolateral incision (parapatellar) was used for fracture exposure. Medial column restoration was performed first with either a medial incision starting on the posteromedial border of the tibial metaphysis or a more central incision centered on the tibial tuberosity. For bicondylar fractures, a double incision was made: one posteromedial (approximately 1 cm from the posterior tibial border) and one anterolateral (peripatellar incision). In both groups, the following standardized surgical steps were taken.

#### *Step 3*

Reduction and stabilization: the knee joint was opened by sub‐meniscal arthrotomy to visualize fracture characteristics and any damage to ligaments and meniscus; reduction of the fragment and temporary fixation was done using K‐wire; depressed fragments were elevated and supported with a compression clamp or temporary K‐wires to obtain anatomical reduction, while bony defects were filled with synthetic tricalcium phosphate or with autologous bone grafts from the iliac crest.

#### *Step 4*

Internal fixation: depending on the fracture patterns, single or dual locking plates (LCP DePuy Synthes) were used to achieve definitive osteosynthesis.

#### *Step 5*

Postoperative protocol: all patients followed the same postoperative protocol and were checked in the same standardized manner by the same trauma team. Postoperative passive and active knee motion was encouraged the day after surgery to stimulate ROM. Toe‐touch weight‐bearing was suggested for 4 to 6 weeks with the use of two crutches, followed by progressive increase to obtain full weight‐bearing at 3 months.

### *Patient Assessment*

Study data collection, as well as radiographic and clinical evaluation, was performed at our institution by three external and independent investigators, who were not involved in the patients' treatment. Patients' characteristics (gender, age at trauma, type of trauma, body mass index [BMI], comorbidities, American Society of Anaesthesiologists (ASA) class to globally estimate surgical risk, and smoking habits) and treatment characteristics (the initial use of an external fixator with a 2 step‐procedure before definitive ORIF, days from trauma to definitive treatment, hospitalization, and complications) were collected by evaluating hospital charts, clinical notes, and surgery reports.

### *Radiographic Outcomes*

#### *Fracture Patterns and Soft Tissue Damage*

**Figure 1:**
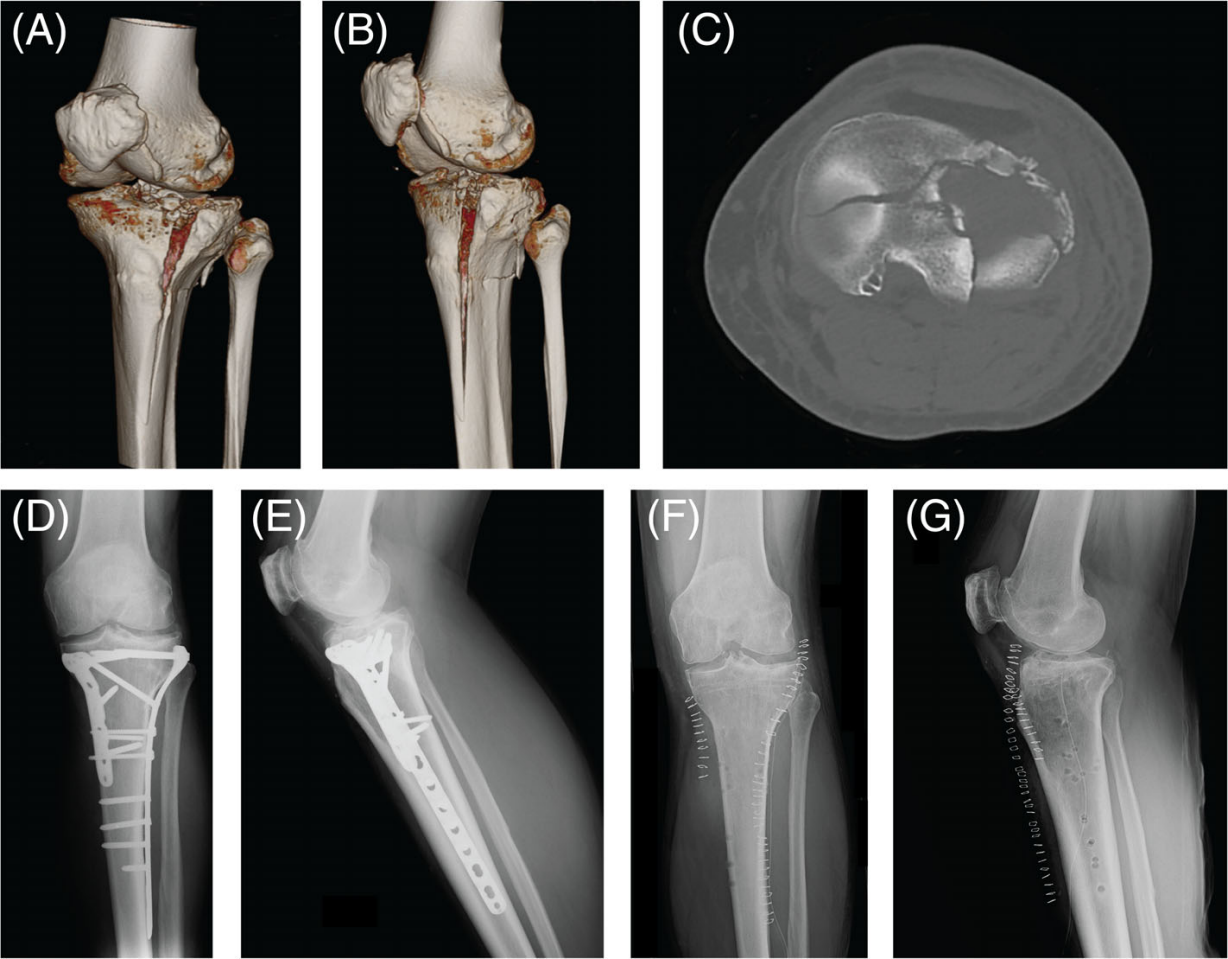
Case 1: A 56‐year‐old man with an AO 41‐C3 fracture treated by open reduction internal fixation (ORIF) technique. (A, B) Preoperative 3D‐CT scan reconstruction images; (C) CT axial image; (D, E) Anteroposterior and lateral radiographic images 6 months after surgery; (F, G) Anteroposterior and lateral radiographic images after removal of implants 35 months after surgery.

**Figure 2:**
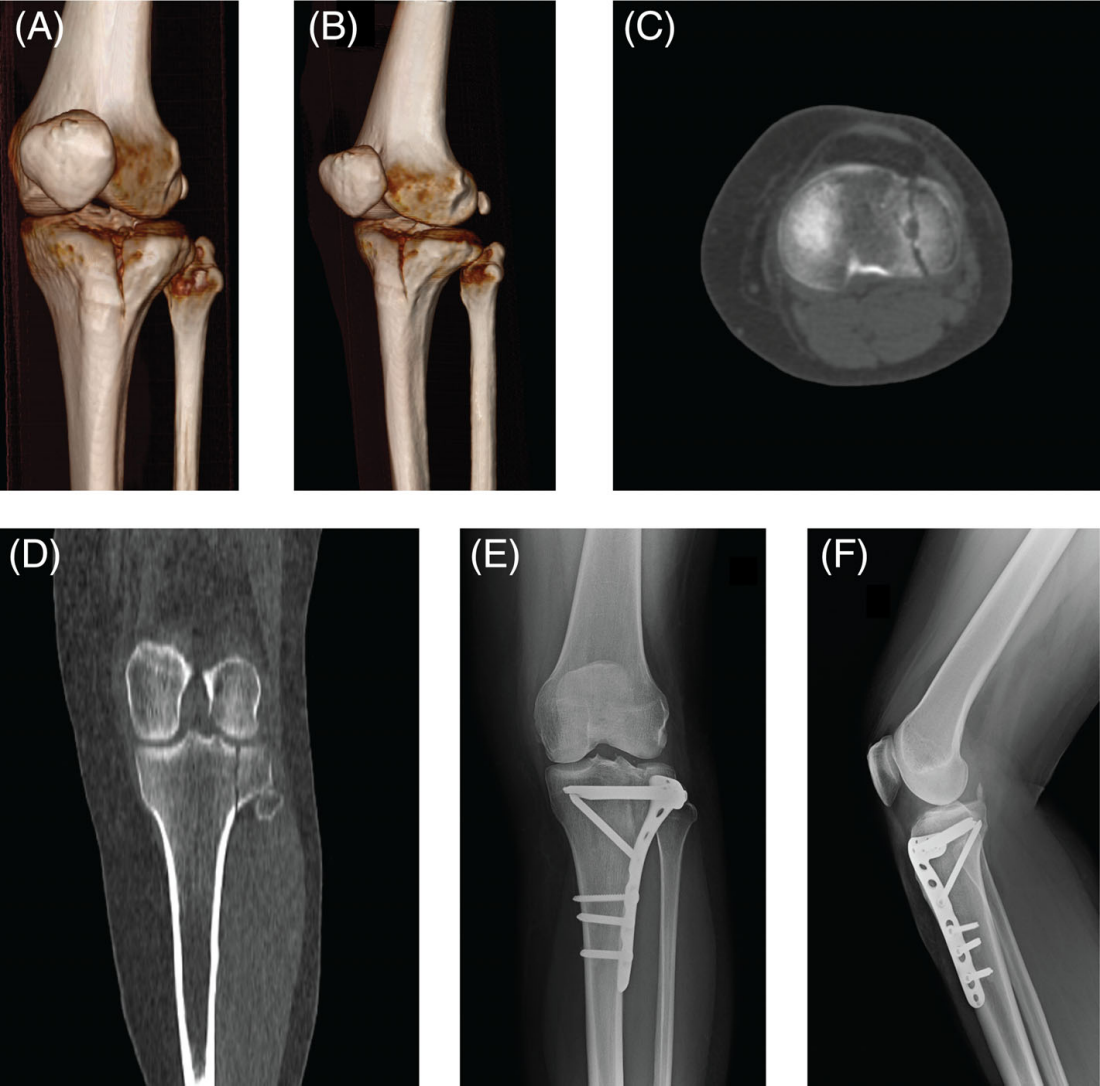
Case 2: A 75‐year‐old woman with an AO 41‐B1 fracture. (A, B) Preoperative 3D‐CT scan reconstruction images; (C) CT axial image; (D) CT coronal view; (E, F) Anteroposterior and lateral radiographic images 6 months after surgery.

**Figure 3:**
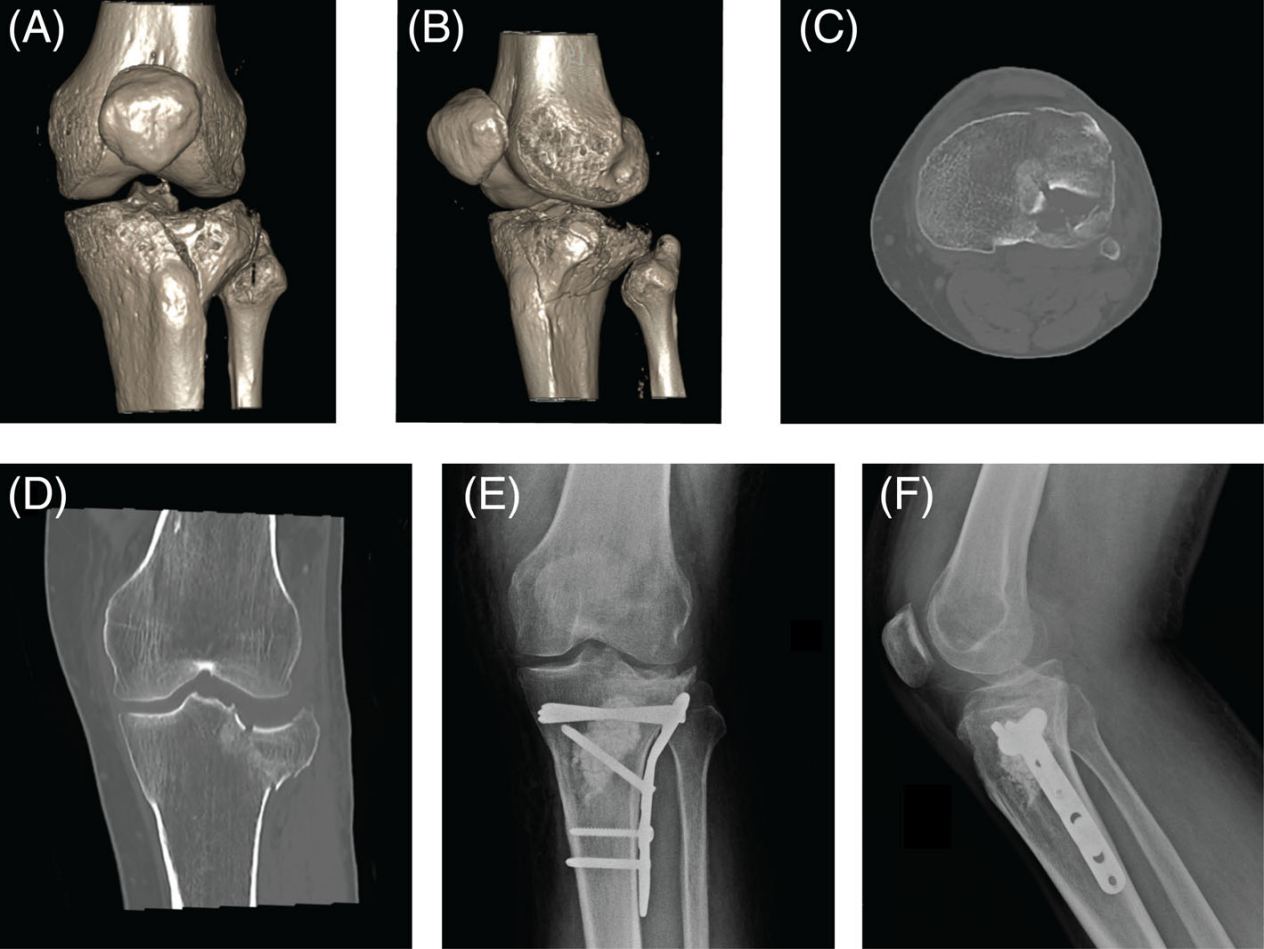
Case 3: A 46 year‐old‐male with an AO 41‐B3 fracture. (A, B) preoperative 3D‐CT scan reconstruction images; (C) CT axial image; (D) CT coronal view; (E, F) Anteroposterior and lateral radiographic images 6 months after surgery made with plate and screws and using artificial bone graft.

Radiographic data were obtained by reading the computerized images available in the X‐ray database computer system (MedStation program) of our institute. Standard radiographs were routinely performed preoperatively, immediately postoperatively, and during the outcome patient examinations 1, 3, 6, 12, and 24 months after surgery, according to our standardized follow‐up protocol. All radiographic evaluations were performed using a diagnostic liquid crystal display (LCD) CORONIS 5MP display (produced by Barco, Rome, Italy) as a viewing monitor to identify fracture patterns and analyze the fractures and their outcomes. The fracture morphology was accurately evaluated and classified into different patterns based on preoperative radiographs and computed tomography (CT) scans (Figs 1, 2, 3). Finally, open fractures and soft tissue damage were also retrieved from the hospital database.

#### *Postoperative Anatomical Reduction and Alignment*

The postoperative reduction was considered anatomic if there was a step‐off ≤1 mm^23^. The malalignment of the fracture was gauged with >5° varus or valgus angulation^26^. These measurements were performed with a simple digital ruler and protractor, included with MedStation Software.

#### *Post‐traumatic Osteoarthritis*

Anteroposterior radiographs of the proximal half of the tibia were evaluated to establish the quality of reduction at 12 months, while, to evaluate the development of PTOA, postoperative radiographs at 24 months were analyzed using the Kellgren–Lawrence scale^27^.

The Kellgren–Lawrence scale is a widely known method to classify the severity of knee OA, analyzing knee radiographs and using five grades (from 0 to 4). In Grade 0, no signs of OA are present, while in Grade 4, there are large osteophytes, as well as marked narrowing joint space, severe sclerosis, and definite bony deformity.

Finally, examination of lateral and anteroposterior view radiographs at different follow‐up points, showing complete bridging bone/callus formation and the absence of radiolucent lines, was used to define bone healing.

### *Clinical Outcomes*

To measure the functional assessment of patients at last follow up, phone contact was attempted for all subjects included in the study, and a follow‐up appointment was fixed. Patients who accepted the follow up were evaluated and clinical results were measured with validated questionnaires (SF‐36, AKSS, KOOS). The visit consisted in a full examination of the lower extremities and gait of all patients.

#### *Range of Motion*

The knee examination included measurement of the ROM of the injured knee, varus‐valgus, and anterior–posterior stability, the presence of a flexion contracture, extension lag, and alignment. The ROM was measured as the passive arc of movement of the examined knee. Measurements were taken with each subject in supine position with the hip flexed on an examination table using a long‐arm goniometer with 50‐cm arms and central articulation. For healthy adult subjects, the normal value of ROM is between 0° and approximately 140° of knee flexion.

#### *Visual Analog Scale*

The VAS is a validated, subjective measure for acute and chronic pain. Scores are recorded by making a handwritten mark on a 10‐cm line that represents a continuum between “no pain” and “extreme pain”^28^. It was used to estimate the pain of the patients.

#### *The Short Form 36*

The SF‐36 questionnaire^29^ is a validated international questionnaire widely used in the current literature to evaluate the physical and mental health of patient. This questionnaire measures general health‐related quality of life and includes 36 questions. All questions are summarized in two different final measures: the physical health status, represented by the Physical Component Summary (PCS), and the mental dimension, represented by the Mental Component Summary (MCS).

The SF‐36 measures eight scales, which contribute in different proportions to the scoring of both PCS and MCS measures. Each scale is scored separately from 0 to 100 points, where lower scores indicate poorer function. These scales are physical function, physical role, bodily pain, general health, vitality, social function, emotional role, and mental health^30^.

#### *American Knee Society Score (Clinical AKSS)*

The American Knee Society Score (AKSS)^31^ is a condition‐specific validated questionnaire widely used to assess knee degeneration problems (OA) and to evaluate postoperative results after total knee replacement. AKSS is an examiner‐dependent clinical evaluation score, which is divided into two independent sections. The first includes clinical evaluation of the knee (clinical AKSS) and the second assesses the patient's functionality in daily life activities (functional AKSS)^32^. Maximum scores of 100 points are possible in each section where higher values correspond to better function. In our study, only the clinical AKSS section was used for patient assessment, which consists of seven items: (i) pain (maximum 50 points); (ii) stability of the knee; (iii) antero‐posterior and medio‐lateral stability (maximum 25 points); and (iv) range of motion (maximum 25 points). The other items, which reduce the score, are the following: (v) flexion contraction; (vi) loss of knee extension; and (vii) poor knee alignment. The maximum score of 100 points is reached when the patient has no pain, with good knee stability and alignment, and at least 125° ROM.

#### *Knee injury and Osteoarthritis Outcome Score*

The knee injury and osteoarthritis outcome score (KOOS)^33^ is a validated score with the purpose of evaluating symptoms and function in subjects with knee injuries and OA. It is a knee‐specific questionnaire, developed to assess the patients' opinion about their knee problems; it is well validated to quantify symptoms and function in subjects with knee injury and OA. The KOOS includes 42 items divided into five separately scored subscales: pain (9 items), other symptoms (7 items), function in daily living (ADL) (17 Items), function in sport and recreation (Sport/Rec) (5 items), and knee‐related quality of life (QoL) (4 items)^33^.

Standardized answer options are used (5 Likert boxes) and all items have five possible answer options scored from 0 (no knee problems) to 4 (severe problems); each of the five scores is calculated as the sum of the items included. Scores are transformed between 0% and 100% using a precise algorithm (100‐mean subscale results/4 × 100)^33^, with 0 representing severe knee problems and 100 representing no knee problems^33^.

#### *Return to Work and Sports Activities*

Finally, a general questionnaire was administered to patients to obtain relevant information for the outcomes of the fracture, such as work and sports activities at the age of the trauma, and their resumption.

### *Statistical Analysis*

Statistical analyses were performed by an independent statistician from our university, using SAS 9.2 (SAS Institute, Cary, NC, USA) for Windows. Categorical variables were reported as number of patients and percentage. Continuous data were checked for a normal distribution with the Shapiro–Wilk test and expressed with mean and standard deviation or median and minimal–maximal values. χ^2^ or Fisher tests were applied to compare categorical variables between groups. The *t*‐test was applied to compare continuous variables between groups. *P*‐values were adjusted for age as a confounder with logistic regression for radiographic outcomes comparing the groups based on the type of fracture. *P*‐values were also adjusted for age as a confounder with ANCOVA for clinical outcomes, again comparing the fracture groups. Univariate analysis to compare clinical with radiographic outcomes was performed with a *t*‐test. Logistic regression analyses were carried out to identify predictors of the three major radiographic outcomes (anatomical reduction, alignment, and Kellgren–Lawrence). Given the small sample size employed, an iterative strategy was used to properly choose the variables to retain in the regression models. An initial cut‐off of 0.25 was adopted, because more restrictive cut‐off points, including the classical threshold of 0.05, could fail in identifying important predictors^35^, ^36^. After running the full model, variables were iteratively deleted until the best reduced regression model was found in terms of the Akaike information criterion (AIC). This method enabled identification of the best predictors for each clinical outcome. Correlations were performed with Pearson's coefficient correlation. Statistical significance was defined for all variables as two‐tailed *P* < 0.05 (α value 5%).

## Results

### *Patient Characteristics*

**Figure 4:**
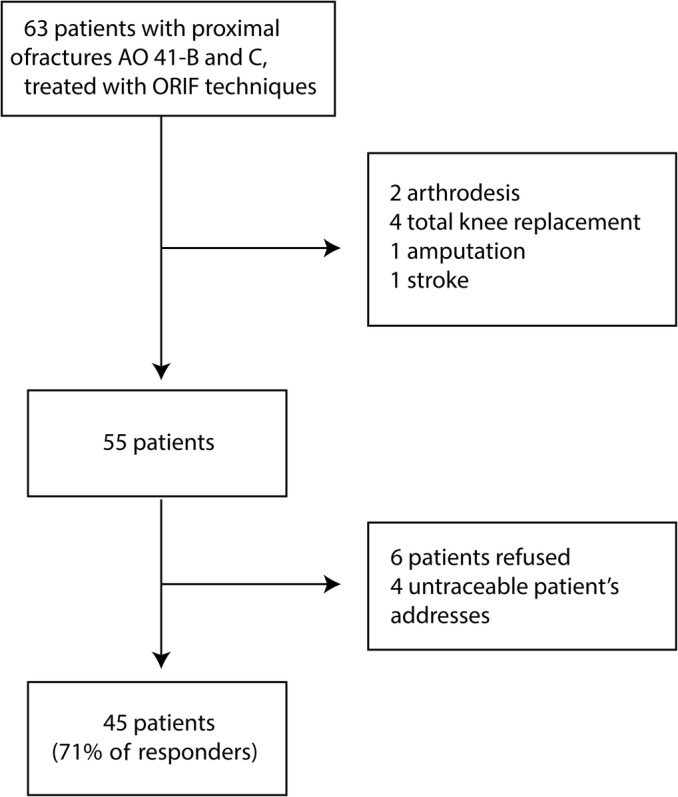
Flow‐chart of patients' selection. ORIF, open reduction internal fixation.

Over a period of 5 years, 55 intra‐articular TPF treated with the ORIF technique in 55 patients fulfilling the inclusion criteria were identified. Among these, we could not evaluate 10 patients: 6 refused to participate, and a follow‐up address could not be retrieved for the other 4 patients (Fig. 4). Therefore, 45 patients were retrospectively enrolled in the present study; no one was lost to the average final clinical follow up of 57.18 months (range, 26–94; SD, 21.72); that is, more than 4 years (Table S1 of the supplementary materials). The mean age at surgery was 54.5 years (range, 24–75); more than half of the participants were men (64.44%), and TPF involved the left knee most frequently (64.44%) (Table 1). Patient comorbidities and risk factors were recorded as well: mean BMI was 26.2 (±4.6) kg/m^2^, one‐third of the patients were active smokers (33.33%), 7 patients (15.56%) had a diagnosis of hypertension, and 9 participants suffered from heart disease (20%) (arrhythmias and previous myocardial infarction). According to the ASA classification of anesthesiological risk, 26 patients were classified as ASA 1 (57.78%), 15 (33.9%) as ASA 2 and 4 (8.89%) as ASA 3.

**Table 1:** Demographic and clinical data of patient series

Indexes	Overall	41‐B	41‐C	*P* value
Number of patients	45	30 (66.7)	15 (33.3)	
Gender, number (%)				0.8257*
Male	29 (64.44)	19 (63.33)	10 (66.67)	
Female	16 (35.56)	11 (36.7)	5 (33.3)	
Age, mean (SD), years	54.5 (12.1)	57.4 (13.1)	49.5 (10.3)	0.040‡
BMI, mean (SD), kg/m^2^	26.2 (4.6)	25.9 (4.6)	26.9 (4.6)	0.532‡
Smokers, number (%)	15 (33.33)	10 (33.33)	5 (33.33)	1.000*
Knee, number (%)				0.8257*
Right	16 (35.56)	11 (36.67)	5 (33.33)	
Left	29 (64.44)	19 (63.33)	10 (66.67)	
Trauma circumstances, number (%)				
Traffic accident	33 (73.33)	20 (66.67)	13 (86.67)	0.1403†
Fall	7 (15.56)	7 (23.33)	0	
Sport trauma	5 (11.11)	3 (10.00)	2 (13.33)	
Trauma, number (%)				0.1526†
High‐energy	40 (88.89)	25 (83.33)	15 (100)	
Low‐energy	5 (11.11)	5 (16.67)	0	
Polytrauma or associated fractures, number (%)	18 (40)	10 (33.33)	8 (53.33)	0.1967*
Soft tissue lesions, number (%)				
None	27 (60)	19 (63.33)	8 (53.33)	0.5186*
Yes	18 (40)	11 (36.67)	7 (46.67)	
Comorbidities, number (%)	16 (35.55)	20 (66.67)	10 (66.67)	1.000*
Surgical procedure, number (%)				
1 step	39 (86.67)	29 (96.67)	10 (66.67)	N/A
2 step	6 (13.33)	1 (3.33)	5 (33.33)	
Complications, number (%)	9 (20.00)	4 (13.33)	5 (33.33)	0.1353†

BMI, body mass index; N/A, not applicable because of the small number of patients; SD, standard deviation.

* χ^2^‐test.

† Fisher test.

‡ *t‐*test.

### *Radiographic Outcomes*

#### *Fracture Patterns and Soft Tissue Damage*

In more than half of the patients (66.67%), AO 41‐B fractures (partial articular fractures) were reported, while the remaining 15 had AO 41‐C fractures (complete articular fractures). The sub‐type AO 41‐B3 was the most common and was reported in 62.22% of patients. In almost all patients (88.89%), high energy trauma was the most common type of injury; traffic accidents were reported in 33 cases (73.33%), followed by falls (15.56%) and sports‐related injuries (11.11%). There was no correlation between AO 41‐B or 41‐C fractures and type of trauma. Concomitant fractures were found in 18 injured patients (40%). Time between trauma and surgery ranged from 1 to 12 days, with a median time of 1 day. The median duration of hospitalization was 7 days (range, 4–48 days). In the present series, 18 patients (40%) were reported to have soft tissue damage. However, only 3 cases (7.67%) presented with associated open fractures: 2 Gustilo grade I and 1 Gustilo grade II. Six cases (13.33%) of TPF were previously stabilized with an axial external fixator (2‐step surgery) before treatment with ORIF techniques (3 open fractures and 3 severe soft tissue damage). AO 41‐C TPF were more frequently stabilized with external fixation with respect to 41‐B (*P* = 0.117). All fractures healed within 3 months following surgery. No significant differences were observed between 41‐B and 41‐C fractures comparing patients' demographic and clinical aspects (Table 1). The only exception was age (*P* = 0.040).

#### *Postoperative Anatomical Reduction and Alignment*

In 24 (53.33%) patients, postoperative radiographs (12 months after surgery) showed anatomical reductions (step‐off ≤1 mm, Table 2). Postoperative alignment at follow up ranged from 10° of varus to 5° of valgus. A non‐optimal alignment at follow‐up was reported in 18 patients (40%, Table 2). Radiographic outcomes were compared between the two groups 41‐B and 41‐C adjusted for age, but no differences were observed.

**Table 2:** Patients' radiographic outcomes (number of patients [%])

Radiographic outcomes	Overall	41‐B	41‐C	*P*	*P* *
Quality of reduction					
No‐anatomical reduction	21 (46.67)	11 (36.77)	10 (66.67)	0.057†	0.1290
Anatomical reduction	24 (53.33)	19 (63.33)	5 (33.33)		
Quality of Alignment					
Malalignment	18 (40)	12 (40.0)	6 (40)	1†	0.8203
Correct Alignment	27 (60)	18 (60.0)	9 (60)		
Kellgren–Lawrence,				0.2157‡	0.0329§
Grade 0	22 (48.9)	17 (56.7)	5 (33.3)		
Grade 1	15 (33.3)	10 (33.3)	5 (33.3)		
Grade 2	6 (13.3)	2 (6.7)	4 (26.7)		
Grade 3	2 (4.4)	1 (3.3)	1 (3.3)		
Grade 4	0	0	0		

* *P* was calculated comparing radiographic outcomes of 41‐B with 41‐C fractures after adjustment for age confounder (logistic regression).

† χ^2^‐test.

‡ Fisher test.

§ Kellgren–Lawrence was divided into two groups: grade 0 and grades 1–4.

#### *Post‐traumatic Osteoarthritis*

Because development of PTOA has been reported after TPF, postoperative radiographs 24 months after surgery were analyzed according to the Kellgren–Lawrence scale (Table 2). We found that 51% of our patients developed signs of PTOA. Specifically, 15 patients were scored as grade 1, 6 as grade 2, and 2 as grade 3. A difference was found comparing patients according to different types of fractures (*P* = 0.0329).

### *Clinical Outcomes*

#### *Range of Motion, Visual Analog Scale, and Short Form 36*

The mean ROM was 117.4° (±10.0°). The VAS reported an average score of 2.9 (±2.2). Mean SF‐36 PCS was 41.4 (±11.2) and mean MCS for mental status was 47.4 (±11.5). These results are summarized in Table 3.

**Table 3:** Patients' clinical outcomes at mean follow‐up of 57.18 months

Clinical outcomes	Overall	41‐B	41‐C	*P* *
ROM, mean (SD)	117.4 (10)	118.7 (8.9)	115.0 (11.9)	0.012
VAS, mean (SD)	2.9 (2.2)	2.8 (2.3)	3.0 (2.0)	0.5150
AKSS score, mean (SD)	79.0 (16.1)	81.8 (15.7)	73.4 (15.8)	0.0166
Excellent, number (%)	23 (51)	17 (56.7)	6 (40.0)	
Good	5 (11)	2 (6.7)	3 (20.0)	
Fair	10 (22)	8 (26.7)	2 (13.3)	
Poor	7 (16)	3 (10)	4 (26.7)	
KOOS score, mean (SD)	69 (20.1)			
Symptoms	77.2 (22.2)	81.2 (19.0)	69.0 (26.3)	0.0807
Pain	82.1 (16.2)	82.6 (16.9)	80.9 (15.1)	0.3984
ADL	82.0 (18.5)	83.1 (20.1)	79.9 (15.1)	0.0914
Sport/Recreation	47.1 (33.5)	47.7 (34.3)	45.7 (33)	0.3257
QoL	55.8 (28.6)	58.9 (30.2)	49.6 (24.8)	0.0243
SF‐36, mean (SD)				
PCS	41.4 (11.2)	42.2 (11.9)	48.3 (11.0)	0.0198
MCS	47.4 (11.5)	39.7 (9.9)	45.6 (12.5)	0.3270

* *P* was calculated comparing radiographic outcomes of 41‐B with 41‐C fractures after adjustment for age confounder (ANOVA).

AKSS, American Knee Society score; KOOS, knee injury and osteoarthritis outcome score; ROM, range of motion in degrees; SD, standard deviation; SF‐36, 36‐Item Short Form Survey; VAS, visual analog score.

#### *American Knee Society Score and Knee Injury and Osteoarthritis Outcome Score*

The AKSS (clinical section) mean score was 79.0 (±16.1), with an overall good outcome. The result of AKSS was “excellent” (score > 80) in 23 patients (51%), “good” (score 70–79) in 5 (11%), “fair” (score 60–69) in 10 (22%), and “poor” (< 60) in 7 (16%). The mean KOOS overall score was 69.0 (±20.1). The mean KOOS was 82.1 (±16.2) for the pain subscale, 77.2 (±22.2) for symptoms, 82.0 (±18.5) for daily function, 47.1 (±33.5) for sports and recreation, and 55.8 (±28.6) for QoL.

#### *Comparison of Preoperative Radiographic Outcomes (Fracture Patterns) with Clinical Outcomes*

After adjustment for age as a confounder, reduced ROM was observed in 41‐C fractures compared to 41‐B fractures (*P* = 0.012). Moreover, AKSS and KOOS QoL were higher for the AO 41‐B fractures compared to AO 41‐C fractures (*P* = 0.0166 and *P* = 0.0243, respectively). In contrast, SF‐36 PCS was higher for 41‐C fractures compared to 41‐B fractures (*P* = 0.0198). No other statistical differences were found evaluating the clinical outcomes between the two groups.

#### *Return to Work and Sport Activities*

Among the 37 patients that were employed at the time of injury, 24 patients (65%) returned to full work capacity. A total of 13 patients (35%) did not return to work or needed to change jobs because of limitation caused by their injury. These patients, compared to those who returned to previous work, showed lower ROM (*P* = 0.019), higher pain measured by VAS (*P* = 0.039), lower clinical scores AKSS (*P* = 0.0006), lower KOOS pain (*P* = 0.001), lower KOOS symptoms (*P* = 0.0083), lower KOOS function (*P* = 0.0008), lower KOOS sports/recreation (*P* = 0.0017), lower KOSS QoL (*P* = 0.0005), and lower SF‐36 PCS (*P* = 0.0002).

In addition, 16 patients (64%) did not return to their previous level of sports activity because of pain. These patients showed higher VAS pain (*P* = 0.02), lower AKSS (*P* = 0.0026), lower KOOS pain (*P* = 0.03), lower KOOS symptoms (*P* = 0.04), lower KOOS function (*P* = 0.02), lower KOOS sports/recreation (*P* = 0.035), lower KOOS QoL (*P* = 0.0674) and lower SF‐36 PCS (*P* = 0.001).

#### *Comparison of Postoperative Radiographic Outcomes with Clinical Outcomes*

Comparing postoperative radiographic outcomes with the clinical outcomes, it was found that patients with a proper anatomical reduction after surgery had a better ROM (*P* = 0.470) and a better outcome in daily life activities (KOOS ADL *P* = 0.0488) and knee function (KOOS QoL *P* = 0.0314; AKSS *P* = 0.0419), and less pain (KOOS pain *P* = 0.0105) compared to patients with a gap or step‐off >1 mm (Table 4). Moreover, patients with a correct knee alignment after surgery displayed higher values of KOOS symptoms and pain subscales compared to the patients with poor knee alignment (*P* = 0.0406 and *P* = 0.0119, respectively) (Table 4). No correlations were found among the number of days of hospitalization and both surgical and clinical outcomes. However, weak negative correlations were found between the number of days of hospitalization and SF‐36 PCS (*r* = −0.3, *P* = 0.0455). Weak negative correlations were observed between BMI and all subscales of KOOS (excluding symptoms subscale) and weak to moderate negative correlations were identified between age and KOOS subscales. Finally, we identified a significant relationship between the presence of PTOA and clinical outcomes at last follow up. In particular, a worse clinical outcome was reported for patients with PTOA compared to patients without PTOA, as demonstrated by a statistical difference in ROM (*P* = 0.0053), AKSS (*P* = 0.021), KOOS sport (*P* = 0.0167), KOOS QoL (*P* = 0.0227), and SF‐36 PCS (*P* = 0.0142), and SF‐36 MCS (*P* = 0.0381) (Table 4).On the basis of univariate analysis (Table 4), logistic regression analyses were performed (Tables 5, 6, 7). It was found that both anatomical reduction and alignment were the best predictors for KOOS pain (*OR* 1.05 [95%*CI* 1.01–1.10], *P* = 0.0182; *OR* 1.05 [1.01–1.10], *P* = 0.0198, respectively) (Tables 5, 6). PTOA was the best predictor for AKSS (*OR* 0.94 [0.90–0.98], *P* = 0.0050) (Table 7).

**Table 4:** Clinical outcomes divided according to radiographic measurements (Mean (SD))

Variable	No‐anatomical reduction (*n* = 21)	Anatomical reduction (*n* = 24)	*P* *
ROM	114.3 (10.6)	120.2 (8.8)	0.0470
VAS	3.4 (2.3)	2.4 (2.1)	0.13
AKSS	73.9 (16.1)	83.6 (15.0)	0.0419
KOOS Pain	75.6 (17.3)	87.7 (13.1)	0.0105
KOOS Symptoms	69.8 (24.1)	83.6 (18.05)	0.0355
KOOS ADL	76.3 (16.3)	87.1 (19.1)	0.0488
KOOS Sport Rec	38.3 (31.0)	54.8 (34.4)	0.0999
KOOS QoL	46.1 (28.5)	64.3 (26.4)	0.0314
SF‐36 PCS	39.4 (12.2)	43.2 (10.2)	0.2631
SF‐36 MCS	45.5 (10.6)	49.1 (12.1)	0.3046

Variable	Malalignment (*n* = 18)	Correct alignment (*n* = 27)	*P* *
ROM	116.1 (9.3)	118.3 (10.6)	0.4731
VAS	3.7 (2.1)	2.4 (2.2)	0.0548
AKSS	74.2 (14.5)	82.3 (16.6)	0.0977
KOOS Pain	74.7 (16.9)	86.9 (14.1)	0.0119
KOOS Symptoms	68.9 (23.7)	82.7 (19.7)	0.0406
KOOS ADL	79.7 (16.6)	83.6 (19.7)	0.4885
KOOS Sport Rec	38.6 (30.0)	52.7 (35.1)	0.1679
KOOS QoL	47.2 (21.6)	61.6 (31.5)	0.0991
SF‐36 PCS	40.3 (10.3)	42.1 (11.9)	0.5994
SF‐36 MCS	45.8 (11.6)	48.5 (11.5)	0.4370

Variable	Kellgren–Lawrence grade 0 (*n* = 22)	Kellgren–Lawrence grade 1–4 (*n* = 23)	*P* *
ROM	121.6 (9.6)	113.5 (9.0)	0.0053
VAS	2.4 (2.3)	3.4 (2.0)	0.1232
AKSS	86.3 (13.1)	72.1 (15.9)	0.0021
KOOS Pain	86.1 (15.4)	78.1 (16.4)	0.0989
KOOS Symptoms	80.3 (21.1)	74.2 (23.3)	0.3675
KOOS ADL	85.4 (19.7)	78.8 (17.0)	0.2309
KOOS Sport Rec	59.1 (33.5)	35.5 (29.9)	0.0167
KOOS QoL	65.6 (27.3)	46.5 (27.1)	0.0227
SF‐36 PCS	45.5 (12.4)	37.5 (8.5)	0.0142
SF‐36 MCS	51.0 (8.6)	44.0 (12.9)	0.0381

* *t*‐test.

AKSS, American Knee Society score; KOOS, knee injury and osteoarthritis outcome score; ROM, range of motion in degrees; SD, standard deviation; SF‐36, 36‐Item Short Form Survey; VAS, visual analog score.

**Table 5:** Logistic regression analyses to identify anatomical reduction as predictor of clinical outcomes

Anatomical reduction	Coefficient	Std. Error	Wald	*P*	*OR*	95% *CI*
Full model						
ROM	0.04	0.06	0.41	0.5228	1.04	0.92 to 1.17
VAS	0.19	0.26	0.51	0.4741	1.21	0.72 to 2.02
AKSS	0.00	0.04	0.00	0.9884	1.00	0.93 to 1.07
KOOS ADL	−0.04	0.05	0.92	0.3384	0.96	0.88 to 1.05
KOOS Pain	0.08	0.05	1.90	0.1680	1.08	0.97 to 1.20
KOOS Symptom	0.01	0.03	0.18	0.6686	1.01	0.96 to 1.06
KOOS QoL	0.01	0.02	0.35	0.5517	1.01	0.97 to 1.06
KOOS Sport Rec	0.00	0.02	0.00	0.9881	1.00	0.97 to 1.03
Constant	−9.17	6.42	2.04	0.1532		
Reduced model						
KOOS Pain	0.05	0.02	5.58	0.0182	1.05	1.01 to 1.10
Constant	−4.26	1.91	5.01	0.0253		

AKSS, American Knee Society Score; KOOS, Knee injury and Osteoarthritis Outcome Score; ROM, range of motion in degrees; SD, standard deviation; SF‐36, 36‐Item Short Form Survey; VAS, visual analog score.

**Table 6:** Logistic regression analyses to identify alignment as predictor of clinical outcomes

Alignment	Coefficient	Std. Error	Wald	*P*	*OR*	95% *CI*
Full model						
VAS	−0.08	0.23	0.12	0.7273	0.92	0.59 to 1.44
AKSS	−0.02	0.04	0.22	0.6372	0.98	0.91 to 1.06
KOOS Pain	0.05	0.04	1.36	0.2428	1.05	0.97 to 1.13
KOOS Symptom	0.01	0.02	0.38	0.5357	1.01	0.97 to 1.06
KOOS QoL	0.00	0.02	0.02	0.8994	1.00	0.96 to 1.04
KOOS Sport Rec	0.00	0.01	0.00	0.9487	1.00	0.97 to 1.03
Constant	−2.77	3.49	0.63	0.4276		
Reduced model						
KOOS Pain	0.05	0.02	5.43	0.0198	1.05	1.01 to 1.10
Constant	−3.75	1.81	4.28	0.0385		

AKSS, American Knee Society Score; KOOS, Knee injury and Osteoarthritis Outcome Score; ROM, range of motion in degrees; SD, standard deviation; VAS, visual analog score; SF‐36, 36‐Item Short Form Survey.

**Table 7:** Logistic regression analyses to identify PTOA as a predictor of clinical outcomes

Kellgren–Lawrence	Coefficient	Standard error	Wald	*P*	*OR*	95% *CI*
Full model						
ROM	−0.16	0.10	2.86	0.0910	0.85	0.70 to 1.03
VAS	−0.33	0.33	1.03	0.3108	0.72	0.38 to 1.37
AKSS	−0.09	0.05	3.40	0.0651	0.91	0.82 to 1.01
KOOS ADL	0.15	0.07	4.74	0.0295	1.16	1.02 to 1.33
KOOS Pain	−0.05	0.06	0.73	0.3920	0.95	0.85 to 1.07
KOOS QoL	0.00	0.03	0.00	0.9851	1.01	0.95 to 1.06
KOOS Sport Rec	−0.01	0.02	0.57	0.4505	0.99	0.95 to 1.02
SF 36 MCS	−0.01	0.04	0.02	0.8916	0.99	0.91 to 1.08
SF 36 PCS	−0.07	0.06	1.32	0.2503	0.94	0.83 to 1.05
Constant	22.89	10.52	4.74	0.0295		
Reduce model						
AKSS	−0.06	0.02	7.88	0.0050	0.94	0.90 to 0.98
Constant	5.13	1.86	7.63	0.0058		

AKSS, American Knee Society score; KOOS, knee injury and osteoarthritis outcome score; ROM, range of motion in degrees; SD, standard deviation; SF‐36, 36‐Item Short Form Survey; VAS, visual analog score.

#### *Complications*

The overall complication rate was 13.33%. There was 1 superficial wound infection, resolved with antibiotics and local wound treatment, and 1 case of deep vein thrombosis (DVT), resolved with 6 months of therapy, and there were 4 cases of transitory deficit of the common peroneal nerve (CPN), which spontaneously resolved in 3 months.

## Discussion

### *Study Aims*

The management of articular TPF remains an important issue for orthopaedic surgeons because of complex fracture geometry and the fractures' often unsatisfactory outcomes and high complication rate^37^. Hence, the purpose of the present retrospective, non‐randomized case series study was to evaluate the early radiographic outcomes and the medium‐term functional outcomes of a consecutive series of patients with displaced AO 41‐B and C TPF treated by ORIF using locking plates. Furthermore, the aim of this report was to investigate whether radiographic features at 12 months after surgery and the development of PTOA at 24 months were predictive of clinical‐functional outcome at mean follow up of 57.18 months (range, 26–94).

We enrolled 45 patients in our study, with a mean age of 54.5 years and mean follow‐up time of 57.18 months. More than half of the patients (66.67%) had partial articular fractures (AO 41‐B), while the remaining subjects (33.33%) had complete articular fractures (AO 41‐C). The mean KOOS score was 69.0, while the mean AKSS and SF‐36 PCS scores were, respectively, 79.0 and 41.4. Significant correlation between age and functional results (KOOS ADL, Sport, QoL, and SF‐36 PCS) and between BMI and KOOS Pain, ADL, Sport, and QoL were found. Complete articular TPF (AO 41‐C) tend to develop PTOA more frequently with respect to partial articular TFP, so the first fracture type had worse clinical outcomes.

Patients with postoperative non‐anatomical reduction with step‐off >1mm and tibial malalignment experienced more pain. Moreover, the presence of PTOA at 2 years follow‐up was predictive of worse AKSS scores.

### *Patients with Tibia Plateau Fractures Treated by Open Reduction Internal Fixation Technique*

Our case series was mainly represented by middle‐aged patients with high‐energy trauma, most of whom were victims of traffic accidents. Although in the past few decades, treatment strategies for high‐energy fractures of the tibial plateau have changed, the traditional ORIF using locking plates with or without previous external fixation allowed us to obtain anatomical and postoperative optimal alignment in half of the patients at last follow‐up.

However, even if an anatomical reduction of the fractures is mandatory (according to Ahearn *et al*.^14^), clinical outcomes of surgically treated TPF depend not only on restoration of alignment or articular surface but also on the condition of soft tissue, in particular ligamentous or meniscal lesions^14^. Hence, evaluation of the condition of the soft tissue is the main factor both for timing and the surgical approach to fixation^38^. In these injuries, soft tissues are often compromised; hence, appropriate preoperative planning and diagnosis can help to reduce the complication rate, in particular wound and implant infections. In the present series, 18 patients (40%) were reported to have soft tissue damage but only 3 presented with associated open fractures. No significant relationship between clinical outcomes and time from trauma to surgery or duration of hospitalization was observed.

### *Radiographic Outcomes*

In our series, even if worsening fracture types resulted in worse outcomes, there was no statistical correlation between radiographic outcomes and fracture patterns. Hence, according to Singleton *et al*.^39^, the articular reduction and alignment seemed to be independent of the fracture type. During the past 20 years, few papers have been published specifically evaluating the radiographic outcomes of articular TPF^8^, ^23^, ^40^, ^41^, and there is still debate regarding their correlation with clinical functional results. By the initial univariate analysis, we found that articular step‐off and knee alignment at 12 months after reduction could influence outcome measures, and this is line with some other studies^18^, ^39^, ^40^. Performing the multivariate analysis, it was found that both anatomical reduction and alignment were the best prognostic factors for KOOS pain but not for other functional knee scores. However, these aspects seem to be partially in line with the current literature. As found by Van Dreumel *et al*., there was no difference in functional outcome measures after anatomical and non‐anatomical reduction (with 1 mm of articular step‐off), even if Van Dreumel *et al*. included in their analysis simpler fractures treated only by screws^23^. In our study, this is probably due to the low value of articular step‐off set to consider the reduction as anatomic. Moreover, Lansinger *et al*. and Volpin *et al*. found that knee stability related to better clinical outcomes rather than the quality of reduction and residual articular step‐off 42, 43. Hence, in these knee injuries, it is essential to obtain joint stability for the restoration of knee articular function, but, at the same time, it is also important to restore the articular surface and knee alignment^18^, ^39^, even if we did not find radiographic measures as predictors of articular function. Furthermore, we found that restoring knee alignment and articular step‐off measured by simple X‐ray were predictive of the pain level perceived by the patients (measured with KOOS Pain), which is one of the most important factors for patient wellness.

The incidence and onset of PTOA after TPF are reported in the literature with a wide range, between 17% to 83% in comminuted fractures^6^, ^11^, ^20^. Our data, in the first univariate analysis, showed that radiographic features were predictive of clinical‐functional outcome at last follow up (AKSS, KOOS Sport, KOOS QoL, and SF‐36). Using multivariate analysis, however, we found that X‐ray signs of OA 24 months after surgery were predictors only of AKSS as a measure of clinical outcome. These data are discussed in the literature; Van Dreumel *et al*. did not find any relationship between radiological OA 1 year after surgery and clinical outcomes because PTOA is a slow process that requires more than 1 year to develop. For this reason, we performed its evaluation at 2 years after surgery and found that it is a significant prognostic factor of lower AKSS score, but it is not a significant predictor of lower KOOS or SF‐36 score. This result is probably due to the difference among questionnaires (AKSS being a clinical score compiled by the physician) and because more time is necessary for the development of a symptomatic osteoarthritic knee. According to our data, half of our patients developed signs of PTOA 2 years after surgery. These patients had worse clinical‐functional outcomes. However, we could not exclude that early OA signs were present before the TPF in adult subjects, but, according to Marsh *et al*., the initial injury can be the most important factor leading to PTOA, despite accurate reduction^8^. Time of follow‐up and type of TPF could affect the outcome, and it will be interesting to evaluate PTOA progression with a long‐term follow‐up and record the percentage of patients who will undergo total knee replacement (TKR). Recently, Parratte *et al*. suggested that TKR should be considered in cases of complex articular fractures of the proximal tibia in elderly patients with symptomatic OA or with poor bone quality that makes internal fixation hazardous^44^.

### *Clinical Outcomes*

Numerous tests for functional outcome after knee injuries have been described in the literature. According to Manidakis *et al*.^20^, our patients had overall good recovery from surgically treated tibial plateau injuries, reporting a mean SF‐36 PCS of 41.4 points, inferior to 50, which is the reference value of the healthy population, but in agreement with the results found by Ahearn *et al*. 14. In our series, good functional results were demonstrated by an AKSS mean score of 79 and an overall mean KOOS score of 69. Our experience was similar to those reported by Van Dreumel *et al*. and Jansen *et al*., even for the more complex AO 41‐C fractures with low complication rate^6^, ^23^. Moreover, in accordance with previous published studies, we found that older age is predictive of a lower functional outcome^11^, ^40^. However, Van Dreumel *et al*. did not find a direct correlation between age and KOOS score^23^. With the available numbers, we also found that BMI is predictive of a lower functional outcome. This factor could be explained by the degenerative microarchitectural changes in tibial subchondral trabecular bone due to obesity, which could limit an optimal degree of remodeling after an injury^45^. Reduced knee ROM after TPF is common; hence, a definitive, stable osteosynthesis is fundamental to allow early joint movement and prevent stiffness. The clinical examination of our patients demonstrated that there was satisfactory functional recovery at mid‐term follow up, with an average ROM of the injured knee of 117.4°, which was similar to values reported in other studies^46^, ^47^.

Finally, TPF have a significant socioeconomic impact, mainly due to time taken off work^20^. In our cohort, 65% of patients returned to work normally, while the other patients were unable to return to their previous jobs or needed to reduce or change their activities. Concerning regular sports activities, only 16 patients (64% of recreational athletic patients) of our series returned to their previous level. Van Dreumel *et al*. reported that one‐third of patients with surgically treated TPF did not return to work after injury, and approximately 50% did not return to their previous sports level^23^. Furthermore, our data showed how working and sports habits were statistically correlated to functional outcomes measured by VAS, AKSS, KOOS, and SF‐36. In particular, persistent pain was significantly related to patients' delay in returning to work and sports activities.

### *Limitations*

Although our study deals with a relevant topic in trauma surgery, several potential limitations may have influenced its results, as it was retrospective and a single‐center investigation. There was possible bias due to the different number of 41‐B and 41‐C TPF included, the varied skill of the different surgeons performing the operations (however, all surgeons in our team have long‐term experience in TPF management), the relatively limited sample size (although comparable to the most recently published report), and the presence of early OA radiographic signs at the time of trauma (even if our series was mainly represented by middle‐aged patients). Moreover, X‐rays were used to evaluate radiographic outcomes. In fact, the measurement of articular step‐off was also performed using knee postoperative X‐rays in other studies 11, 14, 23, 48.

Despite these limitations, our study reported detailed radiographic outcomes at 12‐month and 24‐month follow up, and mid‐term clinical functional outcomes of a consecutive series of patients affected by articular TPF and surgically treated by ORIF. Furthermore, as the development of PTOA in half of our cases was already observed 2 years after trauma, we strongly believe that patients with joint malalignment and/or unsatisfactory anatomical reduction should have a longer follow up, with an associated long‐term rehabilitative program and a chondroprotective therapy to prevent PTOA.

### *Conclusion*

In conclusion, the present study first provides useful data regarding the importance of postoperative radiographic and functional follow up after TPF. Second, it highlights that the early radiographic features (congruent articular reduction and tibial plateau alignment) may be predictive for clinical outcomes in terms of pain felt by patients at mid‐term follow up, and PTOA development may be predictive for knee function measured with AKSS. Finally, it confirms that the challenging management of TPF consists not only in the mere treatment of acute knee damage but also in the effort to limit the consequent clinical, social, economic, and health impact of these severe injuries.

## Supporting information


**Table S1.** Demographic data, fracture type, radiological, and clinical outcome scores of our case series.
